# Ocular trauma score as a visual prognostic factor of open globe injuries in a hospital of southern Brazil

**DOI:** 10.5935/0004-2749.20210095

**Published:** 2025-08-21

**Authors:** Beatriz Gubert Deud, Heloiza Favaro Hammerschmidt, Murilo Varela Kniggendorf, Luisa Moreira Hopker, Guilherme Gubert Müller

**Affiliations:** 1 Faculdade Evangélica Mackenzie do Paraná, Curitiba, PR, Brazil; 2 Serviço de Oftalmologia, Hospital do Trabalhador, Curitiba, PR, Brazil

**Keywords:** Trauma severity indices, Visual acuity, Eye injuries, Prognosis, Índices de gravidade do trauma, Acuidade visual, Traumatismos oculares, Prognóstico

## Abstract

**Purpose:**

To compare the visual acuities predicted by the Ocular Trauma Score and each one of the Ocular Trauma Score variables with the final visual acuities of the victims of open globe injuries in a southern Brazil hospital.

**Methods:**

A total of 120 eyes of 119 individuals with open globe injuries were analyzed in this cross-sectional retrospective study that was developed in a university hospital. The information on age, sex, affected eye, trauma mechanism, and other data (such as initial visual acuity, the presence of globe rupture, perforating injury, endophthalmitis, retinal detachment, and afferent pupillary defect) were used to calculate the Ocular Trauma score, and the final visual acuities of all patients were retrieved from the patients’ medical records.

**Results:**

We noted an agreement between the visual acuity predicted by the Ocular Trauma Score and the final visual acuity in our study. An isolated analysis of the variables demonstrated significance with regard to the initial visual acuity (p<0.001), retinal detachment (p=0.001), and afferent pupillary defect (p=0.004). No significant differences were detected between the final visual acuities and those determined by the Ocular Trauma Score system for the present study population.

**Conclusions:**

The Ocular Trauma Score can be applied for the determination of the visual prognoses of victims of open globe injuries. The most significant variables in this predictive analysis are initial visual acuity, retinal detachment, and afferent pupillary defect. Prospective studies with larger sample sizes are required to confirm our findings.

## Abstract (pt)

**Objetivo:**

Comparar a acuidade visual prevista pelo Escore de Trauma Ocular com a acuidade visual final dos pacientes vítimas de trauma ocular aberto atendidos em hospital no sul do Brasil. **M**é**todos:** Foram analisados 120 olhos de 119 vítimas de trauma ocular aberto. Foi realizado um estudo observacional e retrospectivo em hospital universitário. Foram extraídos dados de prontuários relacionados a idade, sexo, olho acometido e mecanismo de trauma, bem como dados para o cálculo do Escore de Trauma Ocular (acuidade visual inicial, presença de ruptura de globo, perfuração, endoftalmite, descolamento de retina, defeito pupilar aferente) e acuidade visual final.

**Resultado:**

Houve concordância entre a acuidade visual prevista pelo Escore de Trauma Ocular e a acuidade visual final prevista no presente estudo. A análise isolada das variáveis demonstrou significância para acuidade visual inicial (p<0,001), para descolamento de retina (p=0,001) e para defeito pupilar aferente (p<0,004). Não houve diferença significativa entre a acuidade visual final do estudo original do Escore de Trauma Ocular. e na população abordada no presente estudo. **Conclus**ã**o:** O Escore de Trauma Ocular pode ser aplicado à população estudada no presente estudo como ferramenta de determinação do prognóstico visual em vítimas de trauma ocular aberto. As variáveis mais significativas são acuidade visual inicial, descolamento de retina e defeito pupilar aferente. Estudos prospectivos com amostras maiores são necessários para comprovar tal hipótese.

## INTRODUCTION

Ocular trauma is considered to be an important cause of visual loss across the world. These injuries are usually unilateral and restricted to the affected eye^(1,2)^. According to the World Health Organization, ocular trauma has an annual global incidence of approximately 55 million and is therefore considered as a major cause of low visual acuity (VA) and preventable blindness, along with the issues of refractive anomalies, cataract, glaucoma, and diabetic retinopathy^(3,4)^.

Young adults, primarily men, are most often affected by ocular trauma; in fact, trauma is the main cause of ocular morbidity among this age group^(3,5,6)^. In both developed and developing countries, most ocular traumas are caused by traffic accidents and occupation-related accidents, often because of the non-use of preventive measures such as seat belts and personal protective equipment^(7,8)^. Among all ocular traumas, open traumas present with the worst visual prognosis^(1)^.

Open globe injuries have significant socioeconomic impact on the victim, the associated family, and the society in general. Victims of severe ocular injury commonly present with complications such as anxiety and psychological changes, financial loss, loss of careerand work-related opportunities, changes in lifestyle, and permanent changes in their physical appearance^(9,10)^.

Well-planned measures based on epidemiological data are believed to significantly reduce the socioeconomic and personal impacts of ocular trauma. In addition, more accurate visual prognoses are expected to assist specialist physicians in decision-making and in comprehending the patients’ perspectives regarding the goal of treatment^(1,11)^. The Ocular Trauma Score (OTS), which was proposed by Kuhn et al.^(12)^, is a simple tool that can be used to estimate the visual prognoses of patients suffering from ocular trauma by the analysis of initial VA and five other variables. The OTS is an effective and accurate tool for assessing visual prognosis across various mechanisms of ocular trauma^(13-15)^. However, the regional characteristics are believed to influence the variation in the risk factors and their significance. Thus, for promoting the application of this tool, it is important to verify the effectiveness accuracy when applied to a random population.

The objective of the present study was to compare the VA predicted by the OTS and the other five OTS variables and the final VA of the victims of open globe injuries treated at a general hospital in southern Brazil in order to validate the effectiveness of the application of this scoring system to our study population.

## METHODS

A cross-sectional retrospective descriptive analysis was designed based on the analyses and reviews of the medical records of victims of open globe injuries treated at the emergency room of the Hospital Universitário Evangélico Mackenzie. Individuals with full-thickness corneal and/or scleral injuries were considered as the victims of open globe injuries. The subjects of this study included these victims of open globe injuries without any previous treatment and who visited the hospital for primary repair during January 2014 to June 2019. Individuals with previous ocular disease in the affected eye, with a clinical follow-up after the trauma of <6 months, or with missing data considered essential to the study in the medical record were excluded from the study.

The data related to patients’ demographics (age, sex, affected eye, and trauma mechanism) as well as the data required to calculate the score (i.e., the initial VA and final VA as well as the presence of globe rupture, perforating injury, endophthalmitis, retinal detachment, and afferent pupillary defect) were obtained. Final VA was determined as the best-corrected vision after at least 6 months following the trauma.

The results of the measurement of quantitative variables were expressed as means and standard deviations. The frequencies and percent of the data were used to present the categorical variables. The association between the variables and the final VA was assessed using Fisher’s exact test. p<0.05 indicated statistical significance. For tests performed using the same dataset, the level of significance was corrected using the Bonferroni procedure. The data were analyzed by the IBM SPSS Statistics v.20.0 software (Armonk, NY: IBM Corp.).

This study was conducted in accordance with the Declaration of Helsinki and received approval from the local research ethics committee.

## RESULTS

A total of 139 medical records (of 140 eyes) were retrospectively processed. Of the 140 eyes of the victims with open globe injuries, 20 were excluded from the analysis: 3 were eliminated because their clinical follow-up period was <6 months and 17 because of the lack of data in the medical record. Finally, 120 eyes of 119 individuals were analyzed in this study.

The subjects included greater number of men (n=99; 83.2%) when compared to women (n=20; 16.8%). The overall mean age of the subjects was 35.6 ± 19.2 years.

Regarding the trauma mechanism, 84 (70.0%) subjects presented with the issue of penetrations, 27 (22.5%) with ruptures, and 9 (7.5%) with perforations.

The initial VA was ≥20/40 in 2 (1.7%) eyes. Furthermore, 13 (10.8%) eyes had an initial VA between 20/200 and 20/50 and 16 (13.3%) had between 1/200 and 19/200, while 64 (53.3%) eyes had an initial VA classified as light perception (LP) or hand motion (HM) and 25 (20.8%) had it as no light perception (NLP).

Of the OTS variables evaluated during the initial care period, the most frequent was afferent pupillary defect(n=44; 36.7%), followed by globe rupture and retinal detachment (n=27; 22.5% each). Perforating injury occurred in 9 (7.5%) subjects, while endophthalmitis occurred in 6 (5.0%). Table 1 presents the classification of the subjects after the calculation of their scores.

**Table 1:** Ocular trauma score classification

Score	n (%)
1	28 (23.3)
2	39 (32.5)
3	41 (34.2)
4	11 (9.2)
5	1 (0.8)
**Total**	120 (100)

**Figure 1:**
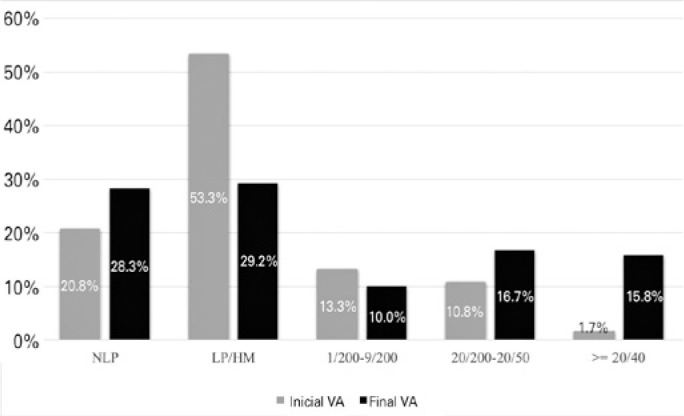
Comparison between the initial and final visual acuity (VA).

The final VA was ≥20/40 in 19 (15.8%) eyes. Furthermore, 20 (16.7%) eyes had a final VA between 20/200 and 20/50 and 12 (10.0%) had a final VA between 1/200 and 19/200, while 35 (29.2%) eyes had a final VA classified as LP or HM and 34 (28.3%) had a final VA classified as NLP. The relation between the initial and final VAs of the subjects is depicted in figure 1.

NLP= no light perception; LP= light perception; HM= hand movement.

An isolated analysis of the variables revealed that the initial VA, retinal detachment, and afferent pupillary defect were significantly associated with worse final VA prognosis. Endophthalmitis, ocular globe rupture, and perforating injury were not considered as significant predictors (Table 2).

**Table 2:** Univariate analysis of the association between risk factors and final visual acuity (%)

Variable	Classification	Final visual acuity		N	p value^*^
		>20/200	<20/200		
Initial visual acuity	<20/200	106	24 (22.6)	82 (77.4)	
	>20/200	14	13 (92.9)	1 (7.1)	<0.001
Rupture	No	93	34 (36.6)	59 (63.4)	
	Yes	27	5 (18.5)	22 (81.5)	0.103
Endophthalmitis	No	114	39 (34.2)	75 (65.8)	
	Yes	6	0 (0)	6 (100)	0.176
Perforation	No	111	37 (33.3)	74 (66.7)	
	Yes	9	2 (22.2)	7 (77.8)	0.716
Retinal detachment	No	93	37 (39.8)	56 (60,2)	
	Yes	27	2 (7.4)	25 (92.6)	0.001
Afferent pupillary defect	No	76	38 (50)	38 (50)	
	Yes	44	1 (2.3)	43 (97.7)	<0.001

* Fisher`s exact test, p<0.05

The significant differences between the final VAs in the present study were calculated and compared with those of the original score. Significant difference was noted between the two populations with regard to the OTS 3 classification alone. No significant differences were noted between the two populations with regard to the other OTS classifications (Table 3).

**Table 3:** Percent of significant differences among cases with visual acuity and OTS final distribution in the present study/OTS study (p value)

OTS	NLP	LP/HM	1/200 - 19/200	20/200 - 20/50	≥20/40
1	75/74 (1)	21/15 (0.407)	0/7 (0.229)	4/3 (0.582)	0/1 (1)
2	32/27 (0.458)	39/26 (0.043)	12/18 (0.514)	10/15 (0.241)	7/15 (0.241)
3	0/2 (0.369)	33/11 (0.003)	17/15 (0.666)	33/31 (0.592)	17/41 (0.003)
4	0/1 (1)	0/2 (1)	0/3 (1)	20/22 (1)	80/73 (0.733)
5	0/0 (1)	0/1 (1)	0/1 (1)	0/5 (1)	100/94 (1)

OTS= Ocular Trauma Score; NLP= no light perception; LP= light perception; HM= hand movement. (Fisher’s exact test, p<0.01)

## DISCUSSION

The profiles of the victims of open globe injuries in the present study demonstrated a prevalence of men (83.2%) of mean age 35.6 years and the predominance of penetration-related injury (70%). The finding of the predominance in penetration injuries conforms to the reports in the literature^(14)^. In addition, a predominance of employment-age men as a risk factor for this type of trauma was noted, considering that the behaviors stereo typically associated with the male gender has influence over of men employed in jobs associated with a higher risk of trauma^(16)^.

Majorly, individuals with an initial VA between 20/200 and LP demonstrated a significant improvement in their final VA. However, from the 25 eyes presenting with initial VAs classified as NLP, 23 had the same final VA; this finding corroborates with those of previous reports in the literature^(12,17,18)^. Another 11 individuals who experienced injury aggravation during their recovery period showed decreased initial VA and remained classified as NLP even at the end of the follow-up period.

When analyzing the relations between the initial and final VA, a significant improvement was noted in subjects with an initial VA >20/200. This finding corroborates with those of Meng et al.^(17)^

Of the variables analyzed, three were specifically significant: initial VA, retinal detachment, and afferent pupillary defect. The initial VA strongly predicts the visual outcome (p<0.001), which again corroborates with the findings of Man et al.^(19)^

Retinal detachment was also a significant predictor (p=0.001), as also corroborated by other studies in the literature^(20-22)^. Man et al.^(19)^ found that retinal detachment was not significant, but they attributed this result to the smaller number of individuals analyzed for this variable. Meng et al.^(17)^ observed that retinal detachment, induced via direct trauma or traction vitreous in open globe lesions, was a significant prognostic factor, probably due to the irreversible lesions in the photoreceptors that reduced the final VA.

The presence of afferent pupilary defect is one of the most significant variables in predicting the visual outcome. If present, this defect indicate a significant damage to the retina or optical pathways with consequent worse prognosis^(1)^. Meng et al.^(17)^ also demonstrated that the presence of afferent pupillary defect could significantly determine visual prognosis, which conforms to the results of the present study (p<0.001). Rofail et al.^(23)^ reported that subjects with afferent pupillary defect are approximately 10-times more likely of have a final VA of ≤20/200, suggesting that this factor is important to predict worse visual prognosis^(23)^.

Open globe rupture, endophthalmitis, and perforating injuries did not significantly predict the visual prognosis (p=0.103, p=0.176, and p=0.716, respectively), most probably because of the small incidence in the sample. As endophthalmitis takes 24-72 h to develop after an open injury, it may not be present at the time of initial examination, and its clinical signs and symptoms may be disguised by the anatomical changes resulting from the injury itself^(24)^. In their study, Man et al.^(19)^ also failed to detect any significance when evaluating the presence of endophthalmitis during the initial care period (p=0.4089); moreover, they did not find any significance with regard to the relation between perforating injury and the final VA (p=0.800).

Although the final visual outcome often remains unclear for weeks after an eye injury, a worse prognosis may depend not only on the preventive measures taken but also on the time gap in initiating treatment. The risk of endophthalmitis may increase after 12 to 24 h of the primary repair post globe rupture. However, past studies suggest that the use of broad-spectrum systemic antibiotics can reduce the risk of infection^(3,17,21)^.

The subjects analyzed in this study were stratified majorly using the same scoring system as used by Kuhn et al.^(12)^, hence the results were comparable with those using the international ocular trauma system (Table 3). Regarding the OTS category 1 that represents the worst visual prognoses, Kuhn et al.^(12)^ reported that 74% of the eyes had a final VA classified as NLP, which is similar to the result of the present study (75% of the eyes). Regarding the OTS category 2, the final VA of the present study was mostly distributed across the categories of NLP (32%), LP/HM (39%), and 1/200-19/200 (12%); these results also corroborate with those based on the OTS system (27%, 26%, and 18%, respectively). Regarding the OTS category 3, the predominant final VAs in the present study were LP/HM and 20/200-20/50 (both 33%), whereas the majority of the eyes analyzed by the OTS system had final VAs of 20/200-20/50 (31%) and ≥20/40 (41%). Regarding the OTS categories 4 and 5, the majority of the eyes presented with final VAs ≥20/40 (80% and 100% in the present study versus 72% and 93% in the OTS study, respectively)^(12)^. The difference results found on the category 3, may suggest that risk factos scores vary when comparing the risk factors from this study to those of the original study. Although larger sample sizes are warranted to confirm this hypothesis.

Overall, we did not find any significant difference in the final VA between the subjects of the present study and those of the study by Kuhn et al.^(12)^, Unver et al.^(14)^, or Sobaci et al.^(25)^ with regard to others OTS categories. This result supports the hypothesis that the tested score may serve as a useful tool in determining the visual prognoses of the victims of open globe injuries^(12,14,25)^.

The small sample size, the possibility of selection bias due to the retrospective study design, the loss to follow-up of certain patients, and the absence of a standardized protocol with regards to patient admission represent some of the limitations of this work. Moreover, the time gap between trauma and treatment, which interferes with the trauma prognosis, was not provided by most medical records and hence could not be considered in the study.

Additional controlled and prospective studies are warranted to clarify the validity of the score obtained in this study for the determination of the visual prognosis.

In conclusion, based on our findings, we believe that the OTS should be applied to determine the visual prognosis of victims of open globe injuries. The most significant variables evaluated in this study included the initial VA and afferent pupillary defect. Our findings indicate that men of mean age 35.6 years are the primary victims of open globe injuries, mostly due to penetrating injuries. However, prospective studies with larger sample sizes are warranted to confirm our findings.
